# Supplementary material for: In vivo diversification of target genomic sites using processive base deaminase fusions blocked by dCas9
Source: Nat Commun. 2020 Dec 22;11:6436. doi: 10.1038/s41467-020-20230-z (PMC7755918; doi:10.1038/s41467-020-20230-z)
Supplement: Supplementary file 1 — Supplementary Information [file 41467_2020_20230_MOESM1_ESM.pdf]

Supplementary information

In vivo diversification of target genomic sites using processive base deaminase fusions blocked by dCas9

Beatriz Álvarez[1], Mario Mencía[2], Víctor de Lorenzo[3] and Luis Ángel Fernández[1*]

[1]Department of Microbial Biotechnology, Centro Nacional de Biotecnología, Consejo Superior de Investigaciones Científicas (CNB-CSIC), Darwin 3, Campus UAM Cantoblanco, 28049 Madrid, Spain. [2]Centro de Biología Molecular "Severo Ochoa" (Consejo Superior de Investigaciones Científicas – Universidad Autónoma de Madrid), Nicolas Cabrera 1, Campus UAM Cantoblanco, 28049 Madrid, Spain. [3]Systems Biology Program, Centro Nacional de Biotecnología, Consejo Superior de Investigaciones Científicas (CNB-CSIC), Darwin 3, Campus UAM Cantoblanco, 28049 Madrid, Spain. *email: lafdez@cnb.csic.es

Supplementary Notes

Expression and activity of different N- and C-terminal fusions of human AID to T7RNAP. AID was fused at the N- and C-terminal ends of T7RNAP using a flexible peptide linker (G3S)7. In addition, variants with N-terminal thioredoxin 1 (TrxA; ~11 kDa) and a cytosolic version of the maltose binding protein (MBPc; ~40 kDa) were also generated (Supplementary Fig. 2a). N-terminal TrxA and MBPc are reported to increase the solubility and expression level of protein fusions in *E. coli* [1, 2]. Native T7RNAP and all AID fusions were expressed in MG*-URA3Δ*ung* under the control of the tetracycline-inducible promoter (TetR-PtetA) [3] in a low copy-number plasmid (pSEVA221) [4]. Bacterial strains in these cultures were grown at 37 °C in LB for 2 h (OD600 ~1.0), and induced with anhydrotetracycline (aTc) for additional 1 h. All cultures grew to a similar final optical density (OD600 ~2.5), except bacteria expressing native T7RNAP (OD600 ~1.2) suggesting some toxicity of T7RNAP expression. Western blot assay of whole-cell protein extracts with a monoclonal antibody (mAb) anti-T7RNAP (Supplementary Fig. 2b), confirmed the presence of protein bands with the expected size for the full-length N-terminal AID fusions but not for the C-terminal fusion (T7RNAP-AID). Also, GFP expression was confirmed by flow cytometry in bacteria expressing native T7RNAP and all N-terminal AID fusions, but not in bacteria expressing the C-terminal fusion of AID or carrying the empty vector (Supplementary Fig. 2c). Therefore, N-terminal fusions to T7RNAP produce a transcriptionally active polypeptide in *E. coli*, whereas C-terminal fusions are not stable and transcriptionally inactive.

To test the mutagenic activity, induced cultures of MG*-URA3Δ*ung* bacteria expressing N-terminal AID fusions, native T7RNAP, or empty vector control, were plated on M9+uracil and M9+uracil+FOA to determine colony forming units (CFU ml^{-1}). The *URA3* mutant frequency was determined for each bacterial strain in three independent experiments as the ratio of FOA^R CFU ml^{-1} *vs*. total CFU ml^{-1} (Supplementary Fig. 2d), revealing a significant increase (~1000-fold) in the frequency of URA3 mutants in cultures expressing N-terminal AID fusions (~10^{-3}) compared to negative control (~10^{-6}). T7RNAP alone increased ~20-fold (~$2x10^{-5}$) the frequency of URA3 mutants, suggesting some mutagenic activity caused by high-level transcription of URA3 and/or by the toxicity of T7RNAP overexpression, which caused a ~10-fold reduction in total CFU ml^{-1} (Supplementary Fig. 2e). In

contrast, bacterial cultures expressing AID fusions had a viability similar to the control strain (~10^9 CFU ml^{-1}) (Supplementary Fig. S2f). Bacteria from the above cultures were also plated on rifampicin (Rif)-containing plates to evaluate the specificity of the off-target mutagenic activity of AID fusions in *rpoB* [5]. This off-target mutation frequency was determined for each culture as the ratio of Rif^R CFU ml^{-1} vs. total CFU ml^{-1}, revealing a ~5-fold increase in *rpoB* mutants in bacteria expressing AID fusions (~10^{-6}) compared to the negative control or bacteria expressing native T7RNAP (~$2x10^{-7}$) (Supplementary Fig. S2f). These data are in accordance with previous work showing that expression of AID slightly increases the mutagenesis of non-specific loci in *E. coli* Δ*ung* strains [6]. However, this small off-target activity was clearly insufficient to explain the ~1000-fold increase in URA3 mutants, indicating a strong on-target specificity of AID fusions.

Supplementary Methods

Plasmids and cloning procedures. Standard methods of amplification, restriction enzyme digestion and ligation were used for most plasmid constructs [7]. The plasmids pGE*pyrF*, pGE*ung* and pGE*nfi* are derived from the *pir*-dependent plasmid pGE (Km^R, R6K origin of replication) [8] with flanking homology regions to delete the genes *pyrF*, *ung* and *nif*, respectively. Thermosensitive plasmids derivatives of pGETS (Km^R, pSC101-ts origins of replication) [9] were used to integrate the mutagenesis reporter cassettes in the non-essential locus of *flu*. The homology regions of the genes *pyrF*, *ung*, *nif* and *flu* were amplified by PCR using as template genomic DNA from *E. coli* MG1655. The gene gfp^{TCD} [10] coding for GFP was obtained from the plasmid pGEyeeJPtac-gfp [9], the URA3 gene was a synthetic version optimized for *E. coli* (GeneCust), the *sacB* gene was amplified from genomic DNA from *E. coli* T-SACK [11], the TEM-1 gene was amplified from the plasmid pBR322 [12]. The proofreading DNA polymerase Herculase II Fusion (Agilent Technologies) was used to amplify DNA fragments for cloning purposes.

Cloning of crRNAs in pdCas9. For cloning the double b.a and the triple b.a.c and d.e.f spacer arrays into pdCas9 to generate the plasmids pdCas9b.a, pdCas9b.a.c and pdCas9d.e.f, respectively, dsDNA fragments containing spacers and direct repeats (DRs) were built using hybridized complimentary oligonucleotides (Supplementary Fig. 8) that are listed in Supplementary Table 3. The sequences of the spacers used can be found in Supplementary Table 4. To generate pdCas9b.a, a dsDNA fragment containing spacer "b"-DR-spacer "a" with *Bsa*I cohesive ends was generated by annealing the pair of oligonucleotides 1 / 2 (Supplementary Fig. 9). To facilitate the cloning procedure, both oligonucleotides were purchased with phosphorylated 5' ends (Merck KGaA). To anneal the oligonucleotides, a mix was prepared in a PCR tube with 1 μl of 100 μM each oligonucleotide (100 pmoles), 2.5 μl of 1 M NaCl and 43 μl of water (Milli-Q). The mix was incubated in a thermocycler with the following annealing program: 1 cycle of 5 min at 95 ºC and 72 cycles of 1 min starting at 95 ºC and decreasing 1 ºC each cycle. The resulting dsDNA solution was kept on ice and diluted 1:10 in Milli-Q water to be used in a ligation mixture that contains 1 μl dsDNA diluted solution (0.4 pmoles), 2 μl pdCas9 digested with *Bsa*I (100 ng), 1 μl (1 U) T4 DNA ligase (Roche Diagnostics GmbH), 2 μl 10X T4 DNA ligase buffer and 14 μl of water (Milli-Q). The mixture was incubated O/N at 8 ºC, and subsequently used to electroporate *E. coli* DH10BT1R. From two resulting Cm^R colonies, plasmid was isolated and the correct cloning of the double spacer array was confirmed by Sanger chain-termination method (Macrogen). The protocol for cloning the triple spacer array into pdCas9 was based on the one-step scheme CRATES (CRISPR Assembly through trimmed end of Spacers) described by Liao *et al.* [13]. In this case, three dsDNA fragments were assembled using the junction sequences GCTG and GAGT (Supplementary Fig. 8). These junction sequences were introduced replacing the first 4 nucleotides at 5' end of the spacers a, c, e and f. This modification does not affect the target specificity, since in the maturation process of the

crRNAs, the spacers are trimmed approximately 10 nucleotides at the 5' end [14]. For building the triple array b.a.c the dsDNA fragments used were: spacer "b"-DR (with annealed oligonucleotides 3 / 4), spacer "a"-DR (with annealed oligonucleotides 5 / 6), and spacer "c" (with annealed oligonucleotides 7 / 8). For the triple array d.e.f the dsDNA fragments used were: spacer "d"-DR (with annealed oligonucleotides 9 / 10), spacer "e"-DR (with annealed oligonucleotides 11 / 12), and spacer "f" (with annealed oligonucleotides 13 / 14) (Supplementary Fig. 8). Each oligonucleotide was 5' phosphorylated as follows: a mix was prepared with 1 μl of 100 μM oligonucleotide (100 pmoles), 5 μl of 10X T4 DNA ligase buffer (Roche Diagnostics GmbH), 1 μl (10 U) of T4 polynucleotide kinase (New England Biolabs) and water (Milli-Q) up to a total volume of 50 μl. The mix was incubated at 37 ºC 30 min, and then, the T4 polynucleotide kinase was inactivated at 65 ºC 20 min. The annealing process was carried out as described above, and 2 μl of each dsDNA fragment (0.2 pmoles) were added to a ligation mix containing 2 μl pdCas9 digested with *Bsa*I (100 ng), 1 μl (1 U) T4 DNA ligase (Roche Diagnostics GmbH), 2 μl 10X T4 DNA ligase buffer and 9 μl of water (Milli-Q). After incubation O/N at 8 ºC, the mix was used to electroporate *E. coli* DH10BT1R cells following standard procedures. From the resulting Cm^R colonies, plasmid was isolated and the correct cloning of the two triple spacer arrays was confirmed by DNA sequencing (Macrogen).

Oligo-mediated allelic replacement method for the generation of MG1655*. The strain MG1655 was transformed with the plasmid pORTMAGE3 (Supplementary Table 2) that harbors the genes *gam*, *bet* and *exo* (λRed recombinase enzymes genes), and the *mutL E32K* (negative dominant variant of *mutL*). Expression of these genes is controlled by the cI857 temperature-sensitive repressor. An O/N culture of MG1655 with pORTMAGE3 was diluted 1:100 in 10 ml LB and grown at 30 ºC until mid-exponential phase (OD600 0.55-0.65),

and then incubated at 42 ºC in a shaking water bath to induce λRed proteins and MutL (E32K) expression for 15 min 250 rpm. The induced culture was placed immediately on ice for at least 5 min, and the cells were pelleted and washed twice with 10 ml ice-cold Milli-Q water. The cells were resuspended in 160 μl of Milli-Q water and kept on ice. Forty microliters of cell suspension were electroporated with 1 μl of 100 μM rph oligo (Supplementary Table 5 and Supplementary Fig 1a). As a control, another 40 μl cell suspension was electroporated with 1 μl TE (10 mM Tris-HCl, 1 mM EDTA, pH 8). Immediately after electroporation, 1 ml of SOC medium (20 g/l Bacto-tryptone, 5 g/l yeast extract, 0.58 g/l NaCl, 0.18 g/l KCl, 2 g/l MgCl2 and 1.2 g/l MgSO4) at room temperature was added and the cell suspensions were transferred to a flask containing 4 ml of SOC. Cells were allowed to recover for 60 min at 30 ºC 250 rpm, after that, 5 ml of LB was added and second cycle of electroporation was performed repeating the previous steps. After the second cycle of oligo recombineering, cells were collected by centrifugation, resuspended in M9 minimal medium and incubated O/N at 30 ºC 250 rpm. The next day, the cultures were diluted 1:10^6 and plated on minimal medium M9 lacking uracil for selection of clones harboring the insertion, since it was described that the modification affected bacterial growth in this medium [15]. After 24 h incubation at 30 ºC, a few larger colonies were observed in the sample that had been electroporated with rph oligo. Only small colonies were observed in the control sample. Twenty-three large colonies were tested for the presence of the extra G base with an allelic-specific PCR using the pairs of primers F_rph_A/R_rph (amplify 200 bp of *rph* allele w/o G) and F_rph_B/R_rph (amplify 200 bp of *rph* allele with G) (Supplementary Table 5). The allelic-specific PCR was carried out using GoTaq Flexi DNA polymerase (Promega) following the manufacturer's instructions. The optimized PCR program in the thermocycler was 1 cycle at 95 ºC for 5 min; 30 cycles at 95 ºC 30 s, 68.4 ºC 30 s and 72 ºC 30 s; and a final cycle at 72 ºC for 7 min. DNA samples from MG1655 and DH10BT1R were used as controls for DNA w/o the extra G and DNA with the

extra G, respectively. Large colonies were tested by this allelic PCR and most of them produced an amplicon for the expected G insertion. This insertion was confirmed in two colonies by PCR amplification with the pair of primers F_seq_pyrE/R_rph, and subsequently by Sanger DNA sequencing (Macrogen) of the amplicons. The modified MG1655 strain was named MG1655*. The sensitivity of MG1655* to 5-FOA in comparison to the parental strain MG1655 was tested plating drops of ten-fold dilutions in 1 X PBS from liquid cultures on minimal medium M9 supplemented with uracil and 5-FOA.

Deletion of the genes *pyrF*, *ung* and *nfi* using a marker-less genome edition strategy. The genes *pyrF*, *ung* and *nfi* were deleted using a strategy based on the generation of double-strand breaks *in vivo* with I-*Sce*I endonuclease. First, the corresponding *E. coli* strain was transformed with the plasmid pACBSR (for expression of I-*Sce*I and λ Red proteins under the control of the P_{BAD} promoter that is inducible with L-arabinose, Supplementary Table 2). Then, the *E. coli* strain with pACBSR was electroporated with the corresponding pGE-based suicide vector (Km^R) carrying the indicated HRs for its integration in the *E. coli* chromosome and I-*Sce*I restriction sites. The resulting Cm^R / Km^R colonies were grown O/N at 37 ºC with shaking (250 rpm) in LB supplemented with Km and Cm. Next day, the cultures were diluted 1:100 in LB supplemented with Cm and incubated at 37 ºC 250 rpm. When the cultures reached the exponential phase of growth (OD_{600} 0.4-0.6), 0.4% (w/v) L-arabinose was added and the cultures were further incubated for 5 h to induce expression of I-*Sce*I and the cleavage of the chromosome at the integration site. This cleavage promoted a second step of homologous recombination that led to the deletion of the vector sequences and the corresponding gene. Individual colonies of the induced cultures were isolated on LB agar plates with Cm. These colonies were streaked on LB agar plates with and without Km to confirm that they are susceptible to Km due to the loss of vector sequences. Using specific primers (Supplementary

Table 3), the gene deletion was assessed by PCR screening. Finally, the plasmid pACBSR was cured from the final strains by passaging the cultures in LB without antibiotic.

Integration of expression cassettes in the *flu* locus using the pGETS plasmids. The *E. coli* strains were transformed with the plasmid pACBSR as above, and the corresponding pGETS plasmid (Km^R) - which contains the HRs for integration in the *flu* locus and the thermosensitive origin of replication pSC101-ts. The $Apra^R$-marker was incorporated in the cassettes to facilitate the selection of the integrants. Individual colonies were grown O/N in liquid LB medium with Km and Cm at 30ºC 250 rpm. Next day, the O/N cultures were diluted 1:100 in LB with Apra and Cm, and incubated in the same conditions until OD_{600} 0.4-0.6. Then, 0.4% (w/v) L-arabinose was added and the temperature was shifted to 37°C to avoid plasmid replication. The cultures were further grown during 5 h and plated on LB agar plates with Apra and Cm. Individual colonies were tested for sensitivity to Km as above and integration of the corresponding cassette was confirmed by PCR using the primer F_flu_int and a specific primer of the cassette (Supplementary Table 3). As above, the plasmid pACBSR was cured from the final strains by passaging the cultures in LB without antibiotic.

Supplementary Table 1. *E. coli* strains used in this study

Strain	Genotype and relevant features	Reference
**DH10B-T1R**	(F- λ-) *mcrA* Δ*mrr-hsdRMS-mcrBC φ80lacZDM15* Δ*lacX74 recA1 endA1 araD139* Δ(*ara, leu*)7697 *galU galK rpsL* (StrR) *nupG tonA*	Novagen
**BW25141**	(F- λ-) Δ*(araD-araB)567*, Δ*lacZ4787*(::rrnB-3), Δ*(phoB-phoR)580*, *galU95*, Δ*uidA3*::*pir*, *recA1*, *endA9*(del-ins)*::FRT*, *rph-1*, Δ*(rhaD-rhaB)568*, *hsdR51*	[16]
**MG1655**	K-12 (F- λ-)	[17]
**MG1655***	MG1655 *rph* (U00096:3815879_3815880insC)[a]	This work
**MG1655*Δ*pyrF***	MG1655* Δ*pyrF*	This work
**MG*-URA3**	MG1655* Δ*pyrF* Δ*flu*::*gfp*-P_{tac}-URA3-P_{T7}-*aac(3)IV*	This work
**MG*-URA3Δ*ung***	MG*-URA3 Δ*ung*	This work
**MG*-URA3Δ*nfi***	MG*-URA3 Δ*nfi*	This work
**MG*-URA3Δ*ung*ΔP_{T7}**	MG1655* Δ*pyrF* Δ*ung* Δ*flu*::*gfp*-P_{tac}-URA3-*aac(3)IV*	This work
**MG*-URA3Δ*ung*Δ*nfi***	MG*-URA3 Δ*ung* Δ*nfi*	This work
**MG*-SacB-URA3Δ*ung*Δ*nfi***	MG1655* Δ*pyrF* Δ*ung* Δ*nfi* Δ*flu*::P_{tac}-*sacB*-*gfp*-P_{tac}-URA3-P_{T7}-*aac(3)IV*	This work
**MG*-SacB-TEM-1Δ*ung*Δ*nfi***	MG1655* Δ*pyrF* Δ*ung* Δ*nfi* Δ*flu*::P_{tac}-*sacB*-*gfp*-P_{tac}-TEM-1-P_{T7}-*aac(3)IV*	This work

Note: (a) Insertion of C between nucleotides 3815879 and 3815880 of genome accession number U00096

Supplementary Table 2. Plasmids used in this study

Plasmid	Relevant features	Reference	Accession number	Hyperlinks
pORTMAGE3	Km^R; pBRRI ori, *cI857*(ts), P_R *exo*, *bet*, *gam*, *mutL E32K*	[18]	Not deposited	Not applicable
pACBSR	Cm^R; p15A ori, P_{BAD}, I-SceI endonuclease and λRed genes	[19]	Not deposited	Not applicable
pBR322	Amp^R, $Tet^{R,}$ pMB1 ori, TEM-1 β-lactamase	[12]	J01749.1	https://www.ncbi.nlm.nih.gov/nuccore/J01749.1/
pGE	Km^R; R6K ori, muticloning site and I-SceI sites	[8]	Not deposited	Not applicable
pGE*pyrF*	Km^R; ca.500 bp homolgy regions flanking *pyrF* cloned in pGE; for deletion of *pyrF*	This work	MN450165	https://www.ncbi.nlm.nih.gov/nuccore/MN450165
pGE*ung*	Km^R; ca.500 bp homolgy regions flanking *ung* cloned in pGE; for deletion of *ung*	This work	MN450166	https://www.ncbi.nlm.nih.gov/nuccore/MN450166
pGE*nfi*	Km^R; ca.500 bp homolgy regions flanking *nfi* cloned in pGE; for deletion of *nfi*	This work	MN450167	https://www.ncbi.nlm.nih.gov/nuccore/MN450167
pGETS	Km^R; pSC101-ts ori, multicloning site and I-SceI sites	[9]	Not deposited	Not applicable
pGETS*flu*URA3	Km^R $Apra^R$; pGETS, *flu* HRs, gfp^{TCD}-P_{tac}-URA3-P_{T7}-*aac(3)IV*	This work	MN450168	https://www.ncbi.nlm.nih.gov/nuccore/MN450168
pGETS*flu*URA3(P_{T7})del	Km^R $Apra^R$; pGETS, *flu* HRs, gfp^{TCD}-P_{tac}-URA3-*aac(3)IV*	This work	MN450169	https://www.ncbi.nlm.nih.gov/nuccore/MN450169
pGETS*flu*SacB-URA3	Km^R $Apra^R$; pGETS, *flu* HRs, P_{tac}-*sacB*-gfp^{TCD}-P_{tac}-URA3-P_{T7}-*aac(3)IV*	This work	MN450170	https://www.ncbi.nlm.nih.gov/nuccore/MN450170
pGETS*flu*SacB-TEM-1	Km^R $Apra^R$; pGETS, *flu* HRs, P_{tac}-*sacB*-gfp^{TCD}-P_{tac}-TEM-1-P_{T7}-*aac(3)IV*	This work	MT795159	https://www.ncbi.nlm.nih.gov/nuccore/MT795159
pSEVA221	Km^R; RK2 ori	[20]	JX560327	https://www.ncbi.nlm.nih.gov/nuccore/JX560327
pSEVA221T7RNAP	Km^R; pSEVA221, *tetR*-P_{tetA}-T7RNAP	This work	MN450171	https://www.ncbi.nlm.nih.gov/nuccore/MN450171
pSEVA221MBPc-AID-T7RNAP	Km^R; pSEVA221, *tetR*-P_{tetA}-MBPc-AID-T7RNAP	This work	MT795157	https://www.ncbi.nlm.nih.gov/nuccore/MT795157
pSEVA221TrxA-AID-T7RNAP	Km^R; pSEVA221, *tetR*-P_{tetA}-TrxA-AID-T7RNAP	This work	MT795158	https://www.ncbi.nlm.nih.gov/nuccore/MT795158
pSEVA221AID-T7RNAP	Km^R; pSEVA221, *tetR*-P_{tetA}-AID-T7RNAP	This work	MN450172	https://www.ncbi.nlm.nih.gov/nuccore/MN450172
pSEVA221pmCDA1-T7RNAP	Km^R; pSEVA221, *tetR*-P_{tetA}-pmCDA1-T7RNAP	This work	MN450173	https://www.ncbi.nlm.nih.gov/nuccore/MN450173
pSEVA221rAPOBEC1-T7RNAP	Km^R; pSEVA221, *tetR*-P_{tetA}-rAPOBEC1-T7RNAP	This work	MN450174	https://www.ncbi.nlm.nih.gov/nuccore/MN450174
pSEVA221TadA*-T7RNAP	Km^R; pSEVA221, *tetR*-P_{tetA}-TadA*-T7RNAP	This work	MN450175	https://www.ncbi.nlm.nih.gov/nuccore/MN450175
pdCas9	Cm^R; p15A ori, *tracrRNA, cas9*(D10A, H840A)*,* repeat-*Bsa*I spacer-repeat	[21]	Not deposited	Not applicable
pdCas9b.a	Cm^R; double spacer array b.a cloned in pdCas9	This work	MW221488	https://www.ncbi.nlm.nih.gov/nuccore/MW221488
pdCas9b.a.c	Cm^R; triple spacer array b.a.c cloned in pdCas9	This work	MW221489	https://www.ncbi.nlm.nih.gov/nuccore/MW221489
pdCas9d.e.f	Cm^R; triple spacer array d.e.f cloned in pdCas9	This work	MW221490	https://www.ncbi.nlm.nih.gov/nuccore/MW221490

Supplementary Table 3. Oligonucleotides used in this study

Name	Sequence (5'-3')	Function
F_bDRa	AAACAAATGAATTTCAGGGTCAGTTTGCCGTACGGTTTTAGAGCTATGCTGTTTTGAATGGTCCCAAAACATAACGTCAC CGTCCAGTTCCACCAGAATG	For generation of the spacer arrays
R_bDRa	AAAACATTCTGGTGGAACTGGACGGTGACGTTAATGTTTTGGGACCATTCAAAACAGCATAGCTCTAAAACCGTACGGCA AACTGACCCTGAAATTCATTT	For generation of the spacer arrays
F_bDR	AAACAAATGAATTTCAGGGTCAGTTTGCCGTACGGTTTTAGAGCTATGCTGTTTTGAATGGTCCCAAAACGCTG	For generation of the spacer arrays
R_bDR	GTTTTGGGACCATTCAAAACAGCATAGCTCTAAAACCGTACGGCAAACTGACCCTGAAATTCATTT	For generation of the spacer arrays
F_aDR	ACGTCACCGTCCAGTTCCACCAGAATGTTTTAGAGCTATGCTGTTTTGAATGGTCCCAAAAC	For generation of the spacer arrays
R_aDR	ACTCGTTTTGGGACCATTCAAAACAGCATAGCTCTAAAACATTCTGGTGGAACTGGACGGTGACGTCAGC	For generation of the spacer arrays
F_c	GAGTGATTGCGTGCTCAGGTAATGATTGTCG	For generation of the spacer arrays
R_c	AAAACGACAATCATTACCTGAGCACGCAATC	For generation of the spacer arrays
F_dDR	AAACCGGTAACACCGTGAAACTGCAGTACAGCGCGTTTTAGAGCTATGCTGTTTTGAATGGTCCCAAAACGCTG	For generation of the spacer arrays
R_dDR	GTTTTGGGACCATTCAAAACAGCATAGCTCTAAAACGCGCTGTACTGCAGTTTCACGGTGTTACCG	For generation of the spacer arrays
F_eDR	GGTGTGGTGGGTCCAGGTATCGTTTCGTTTTAGAGCTATGCTGTTTTGAATGGTCCCAAAAC	For generation of the spacer arrays
R_eDR	ACTCGTTTTGGGACCATTCAAAACAGCATAGCTCTAAAACGAAACGATACCTGGACCCACCACACCCAGC	For generation of the spacer arrays
F_f	GAGTGCGAAATCCGACAAAGACTTCGTTATG	For generation of the spacer arrays
R_f	AAAACATAACGAAGTCTTTGTCGGATTTCGC	For generation of the spacer arrays
rph	AGAGCTACTCATCTTGTTGGCTCTGGCCCGAGGGGGAATCGAATCCATTGTAGCGACGCAGAAGGCGGCG	Allelic replacement method
F_rph_A	CGCTACAATGGATTCGATTCCCCT	Allelic replacement method
F_rph_B	GCTACAATGGATTCGATTCCCCC	Allelic replacement method
R_rph	GTCGGAATTGTGAACGGCGAAG	Allelic replacement method
F_seq_pyrE	GCCTAACAGTGCCAGATCGCG	Allelic replacement method
F_pyrF_del	GAGAACACCGGTGCCGATGC	Confirmation of *pyrF* deletion by PCR
R_pyrF_del	GGTGCAGTACTGACCGATGGCAGTAC	Confirmation of *pyrF* deletion by PCR
F_ung_del	CGCGACGCAGAACGTTAACGTTC	Confirmation of *ung* deletion by PCR
R_ung_del	CAACCGTGCAGCAGTGGGTAAG	Confirmation of *ung* deletion by PCR
F_nfi_del	GATGTATGCCGATCCGCAGGC	Confirmation of *nfi* deletion by PCR
R_nfi_del	GTCCCATTCTTGCTCTACAGCTGGG	Confirmation of *nfi* deletion by PCR
F_flu_int	CGGTTCACAGGCAATTGGCGGTATTGTTAAC	Confirmation of cassette integration in *flu* locus
R_T1_Ter	GGTGCCCGGGGGCATCAAATAAAACGAAAGGCTCAG	Confirmation of *gfp-URA3* cassettes integration in *flu* locus
R_sacB	CTCCTGCCAGCAGTGCGGTAG	Confirmation of *sacB-gfp-URA3* and *sacB-gfp-TEM-1* cassettes integration in *flu* locus
F_rpoB	AGAGCGTGCGGTGAAAGAGCG	PCR and Sanger sequencing
R_rpoB	CATCCACGTACTGAACGACACCA	PCR and Sanger sequencing
F_GFPseq	GTACGTGGCGTCACCTTCACCC	PCR and Sanger sequencing
R_T0ter	TACGAAGCTTCTGGATTCTCACCAATAAAAAACGCC	PCR and Sanger sequencing
F_CS1_URA3	ACACTGACGACATGGTTCTACAGGTGTGGTGGGTCCAGGTAT	PCR and Illumina sequencing
R_CS2_URA3	TACGGTAGCAGAGACTTGGTCTCGGCGTCATAATCAGCCAAT	PCR and Illumina sequencing

Supplementary Table 4. Oligonucleotides to generate the spacer arrays

Number	Name	Sequence (5'-3')
**1**	F_bDRa	AAACAAATGAATTTCAGGGTCAGTTTGCCGTACGGTTTTAGAGCTATGCTGTTTTGAATGGTCCCAAAACATAACG TCACCGTCCAGTTCCACCAGAATG
**2**	R_bDRa	AAAACATTCTGGTGGAACTGGACGGTGACGTTAATGTTTTGGGACCATTCAAAACAGCATAGCTCTAAAACCGTA CGGCAAACTGACCCTGAAATTCATTT
**3**	F_bDR	AAACAAATGAATTTCAGGGTCAGTTTGCCGTACGGTTTTAGAGCTATGCTGTTTTGAATGGTCCCAAAAC**GCTG**
**4**	R_bDR	GTTTTGGGACCATTCAAAACAGCATAGCTCTAAAACCGTACGGCAAACTGACCCTGAAATTCATTT
**5**	F_aDR	ACGTCACCGTCCAGTTCCACCAGAATGTTTTAGAGCTATGCTGTTTTGAATGGTCCCAAAAC
**6**	R_aDR	**ACTC**GTTTTGGGACCATTCAAAACAGCATAGCTCTAAAACATTCTGGTGGAACTGGACGGTGACGT**CAGC**
**7**	F_c	**GAGT**GATTGCGTGCTCAGGTAATGATTGTCG
**8**	R_c	AAAACGACAATCATTACCTGAGCACGCAATC
**9**	F_dDR	AAACCGGTAACACCGTGAAACTGCAGTACAGCGCGTTTTAGAGCTATGCTGTTTTGAATGGTCCCAAAAC**GCTG**
**10**	R_dDR	GTTTTGGGACCATTCAAAACAGCATAGCTCTAAAACGCGCTGTACTGCAGTTTCACGGTGTTACCG
**11**	F_eDR	GGTGTGGTGGGTCCAGGTATCGTTTCGTTTTAGAGCTATGCTGTTTTGAATGGTCCCAAAAC
**12**	R_eDR	**ACTC**GTTTTGGGACCATTCAAAACAGCATAGCTCTAAAACGAAACGATACCTGGACCCACCACACC**CAGC**
**13**	F_f	**GAGT**GCGAAATCCGACAAAGACTTCGTTATG
**14**	R_f	AAAACATAACGAAGTCTTTGTCGGATTTCGC

Spacers b and d are shadowed in green, a and e in cyan, and c and f in yellow. DR nucleotides are underlined and the connector sequences are in red.

Supplementary Table 5. Target complementary sequences in the spacers.

Spacer	Sequence (5'-3')
a	ACGTCACCGTCCAGTTCCACCAGAAT
b	GAATTTCAGGGTCAGTTTGCCGTACG
c	GATTGCGTGCTCAGGTAATGATTGTC
d	AACACCGTGAAACTGCAGTACAGCGC
e	GGTGTGGTGGGTCCAGGTATCGTTTC
f	GCGAAATCCGACAAAGACTTCGTTAT

Supplementary Table 6. Oligonucleotides used in the allelic replacement method

Name	Sequence (5'-3')	Use
rph	A*G*AGCTACTCATCTTGTTGGCTCTGGCCCGAGGGG**G**AATCGAATCCATTGTAGCGACGCAGAAGGCGG*C*G	G insertion in *rph*
F_rph_A	CGCTACAATGGATTCGATTCCCCT	Allelic-specific PCR
F_rph_B	GCTACAATGGATTCGATTCCCCC	Allelic-specific PCR
R_rph	GTCGGAATTGTGAACGGCGAAG	Allelic-specific PCR
F_seq_pyrE	GCCTAACAGTGCCAGATCGCG	Amplification and sequencing of *pyrE*

(*) Asterisks indicate phosphorothioate bonds.

Supplementary Table 7. Oligonucleotides used for Sanger and Illumina DNA sequencing.

Name	Sequence (5'-3')
F_rpoB	AGAGCGTGCGGTGAAAGAGCG
R_rpoB	CATCCACGTACTGAACGACACCA
F_GFPseq	GTACGTGGCGTCACCTTCACCC
R_T0ter	TACGAAGCTTCTGGATTCTCACCAATAAAAAACGCC
F_CS1_URA3	ACACTGACGACATGGTTCTACAGGTGTGGTGGGTCCAGGTAT
R_CS2_URA3	TACGGTAGCAGAGACTTGGTCTCGGCGTCATAATCAGCCAAT

The Illumina tag sequences CS1 and CS2 are underlined.

Supplementary Figure 1

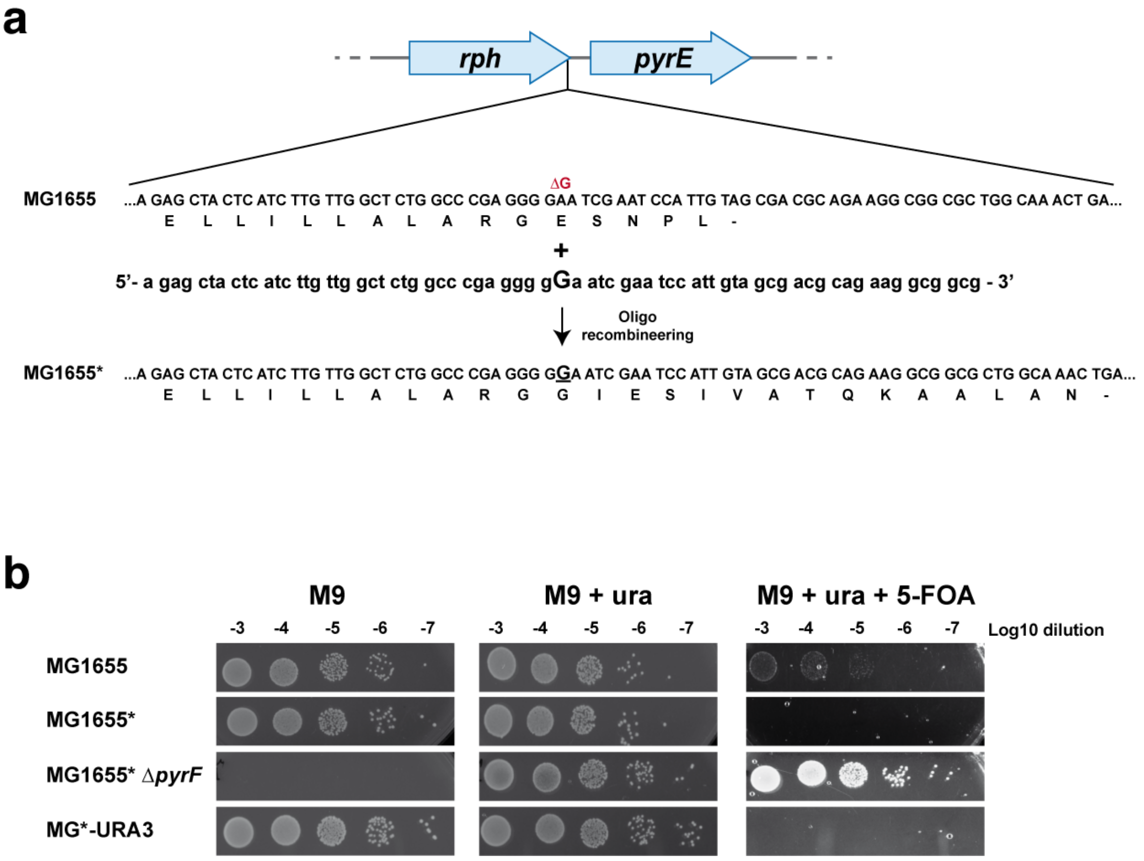

Supplementary Figure 1. Generation and characterization of the reporter strain MG*-URA3. (**a**) Scheme of the operon with the genes *rph* and *pyrE* from MG1655. It is shown the DNA sequence at the 5' end of *rph* with its amino acid translation below and the oligonucleotide used for G insertion resulting in the strain MG1655*. The genes *rph* and *pyrF* are represented with filled arrows. (**b**) Viability of different strains in minimal medium M9, M9 supplemented with uracil and M9 supplemented with uracil and 5-FOA. Series of ten-fold dilutions of each culture were prepared with 1X PBS and 10 μl drops of each dilution were plated. Source data are provided as a Source Data file.

Supplementary Figure 2

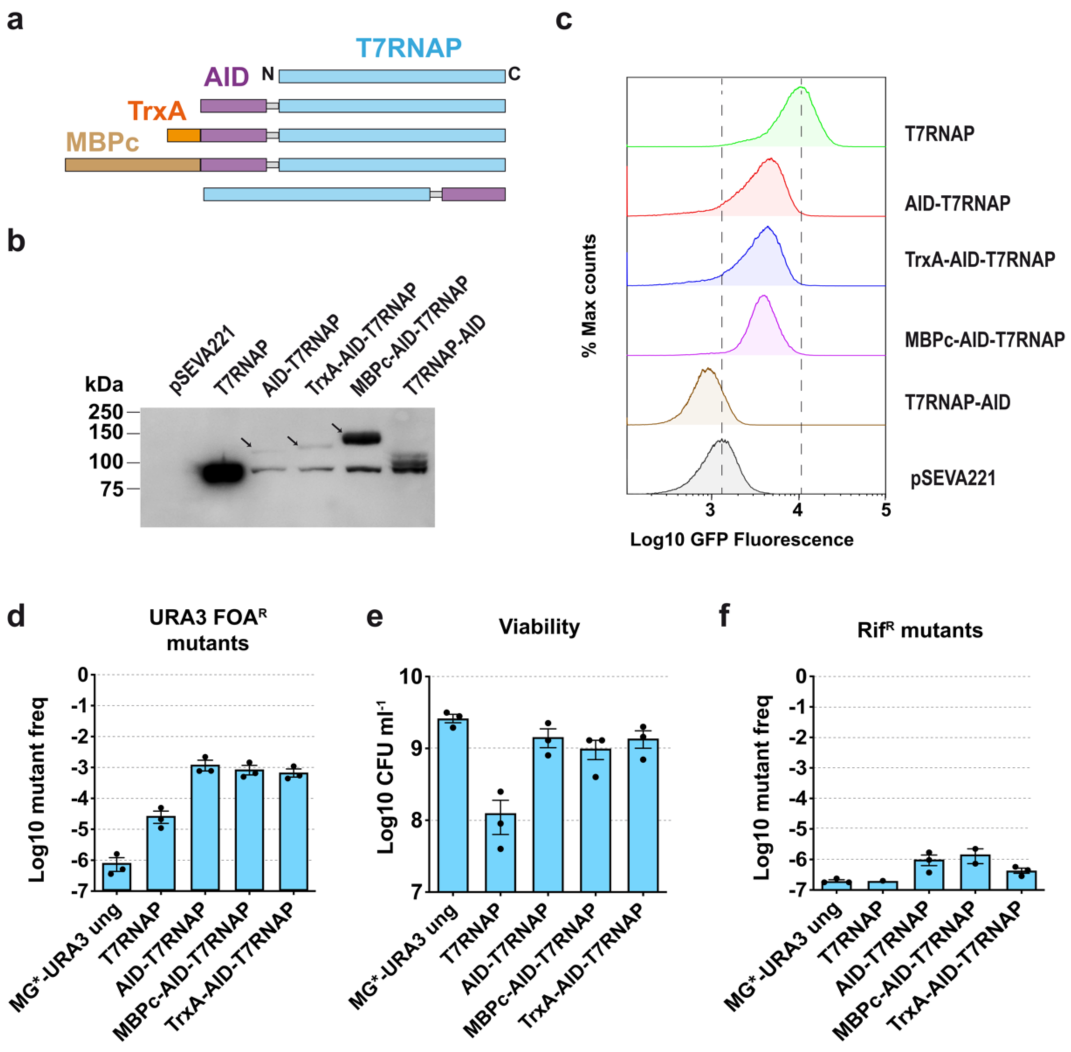

Supplementary Figure 2. Expression and activity of the different variants of the fusion AID-T7RNAP. (**a**) Representation of the different AID fusion variants. (**b**) Expression of the different variants determined by Western blot analysis of the cell extracts from induced cultures of the strain MG*-URA3Δ*ung* transformed with the different plasmids. The arrows indicate the bands corresponding to the full-length fusions. A representative experiment was shown from three independent experiments with similar results (**c**) Processivity of the fusions assessed by flow cytometry analysis to detect expression of *gfp* in the induced cultures. The gating strategy and the corresponding pseudocolor plots are shown in Supplementary Fig. 3a. (**d**) *URA3* mutation frequency of as the ratio of FOA^R CFU ml^{-1} *vs*. total CFU ml^{-1}. (**e**) Viability as Log_{10} CFU ml^{-1}. (**f**) Mutation frequency of *rpoB* as the ratio of Rif^R CFU ml^{-1} *vs*. total CFU ml^{-1}. The histograms (**d**, **e** and **f**) show the single values (black dots), the means (bars) and standard errors (lines) of three independent experiments (n=3). Source data are provided as a Source Data file.

Supplementary Figure 3

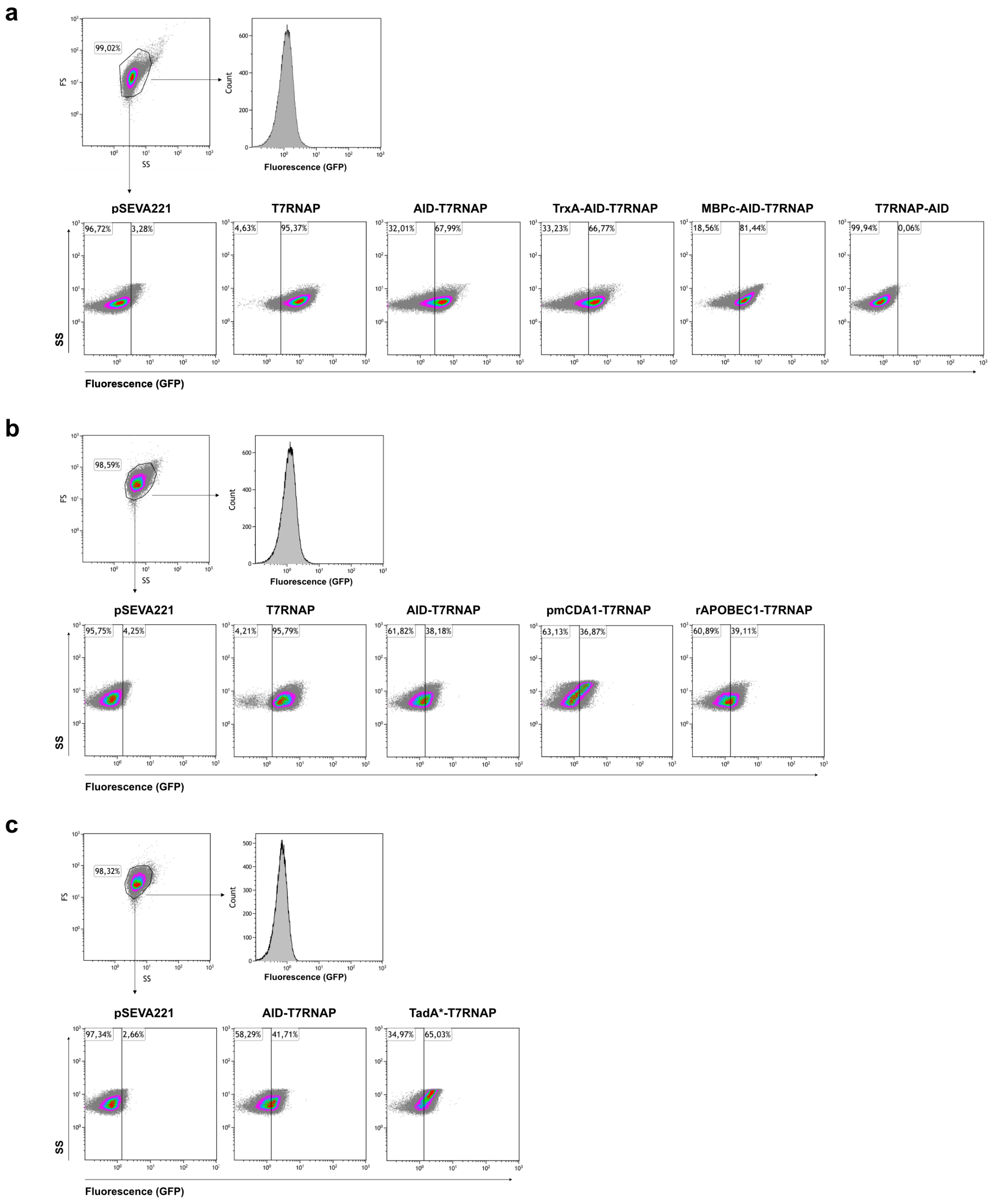

Supplementary Figure 3. Gating strategy used for flow cytometry analysis and pseudocolor plots of the gated bacteria. Gating strategy of the flow cytometry analysis to detect expression of *gfp* in the Supplementary Fig. 2c (**a**), Fig. 2c (**b**) and Fig. 3c (**c**). In each case, a forward scatter (FS) vs. side scatter (SS) dot blot is shown with the gate to select the bacteria for fluorescence determination. Side scatter (SS) vs. fluorescence (GFP) pseudocolor plots show the gated bacteria with the percentages of fluorescence positive and negative bacteria in relation to control cultures with empty plasmid pSEVA221.

Supplementary Figure 4

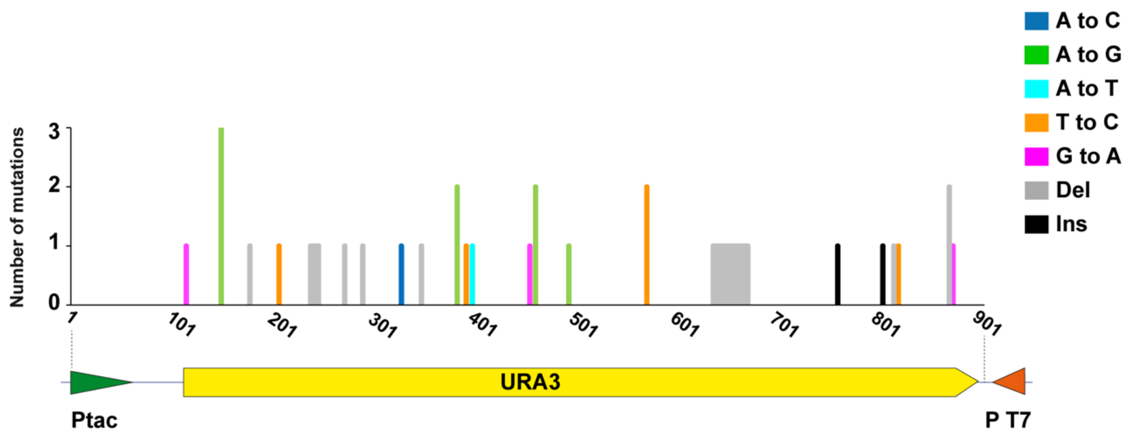

Supplementary Figure 4. Characterization of URA3 mutations in 30 FOA^R colonies from strain MG*-URA3Δ*ung* expressing native T7RNAP. The position of the tac and T7 promoters are indicated (arrow heads). The identified mutations (following the color code on the right) are indicated with respect to the coding strand of URA3 (yellow filled arrow). Del: deletion; Ins: insertion. Source data are provided as a Source Data file.

Supplementary Figure 5

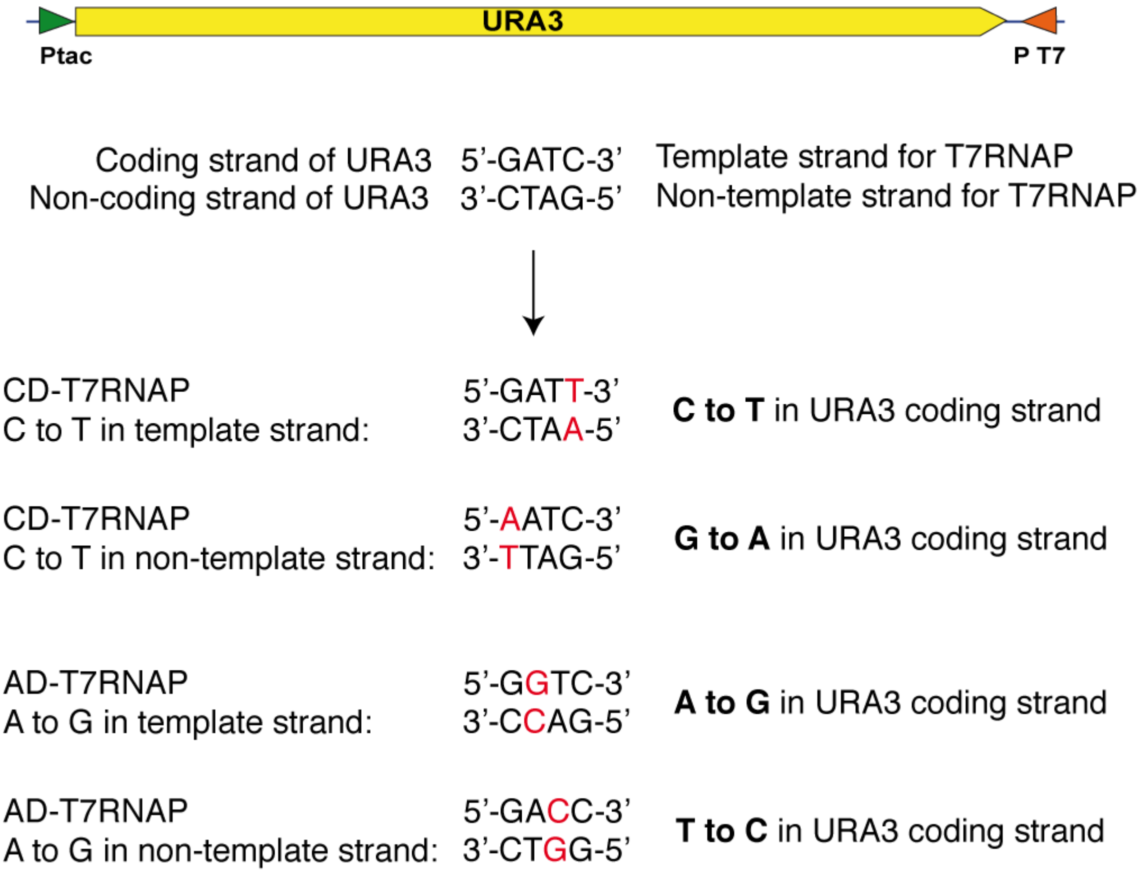

Supplementary Figure 5. Orientation of the DNA strands of URA3 in relation to the tac and T7 promoters. The Ptac and T7 promoters are indicated with arrow heads, and the *URA3* gene with a yellow filled arrow.

Supplementary Figure 6

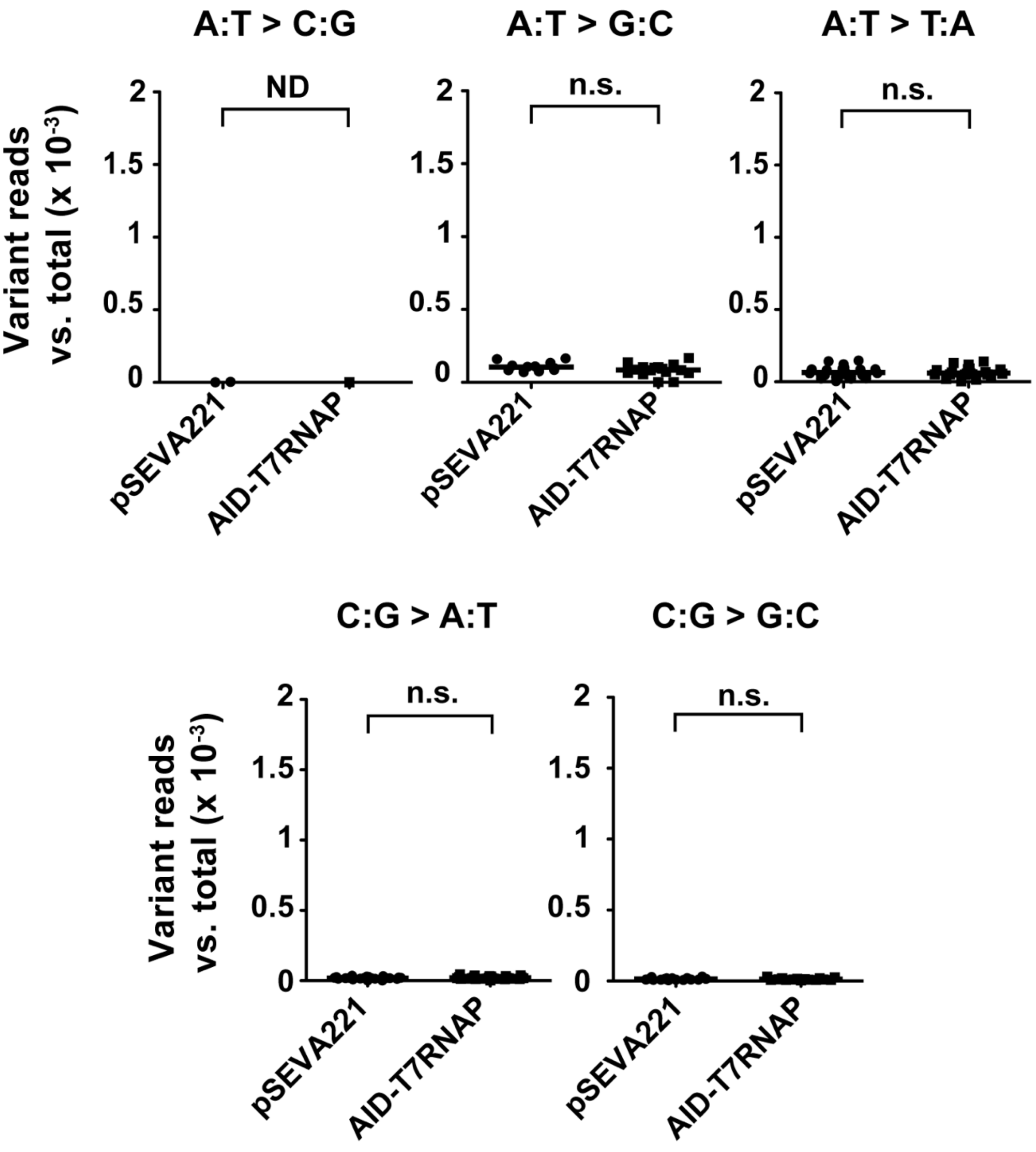

Supplementary Figure 6. High throughput sequencing of URA3 mutated with AID-T7RNAP. Variant calling analysis of a 200 bp region of URA3 after its massive DNA sequencing (ca. 10^6 reads) upon amplification from the strain MG*-URA3Δ*ung* with the empty plasmid (pSEVA221) or expressing AID-T7RNAP. The number of reads with different variants *vs*. total reads are represented with circles (empty plasmid) and squares (AID-T7RNAP). The number of variants found per group (n) in each graph was: A:T>C:G (pSEVA221 n=2, AID-T7RNAP n=1), A:T>G:C (pSEVA221 n=14, AID-T7RNAP n=16), A:T>T:A (pSEVA221 n=25, AID-T7RNAP n=25), C:G>A:T (pSEVA221 n=25, AID-T7RNAP n=27), C:G>G:C (pSEVA221 n=16, AID-T7RNAP n=15).The lines represent the means and the standard errors from each group. The statistical analysis was done using two-tailed Mann Whitney test. ND, not determined for groups with less than three variants detected, n.s. (not significant). Source data are provided as a Source Data file.

Supplementary Figure 7

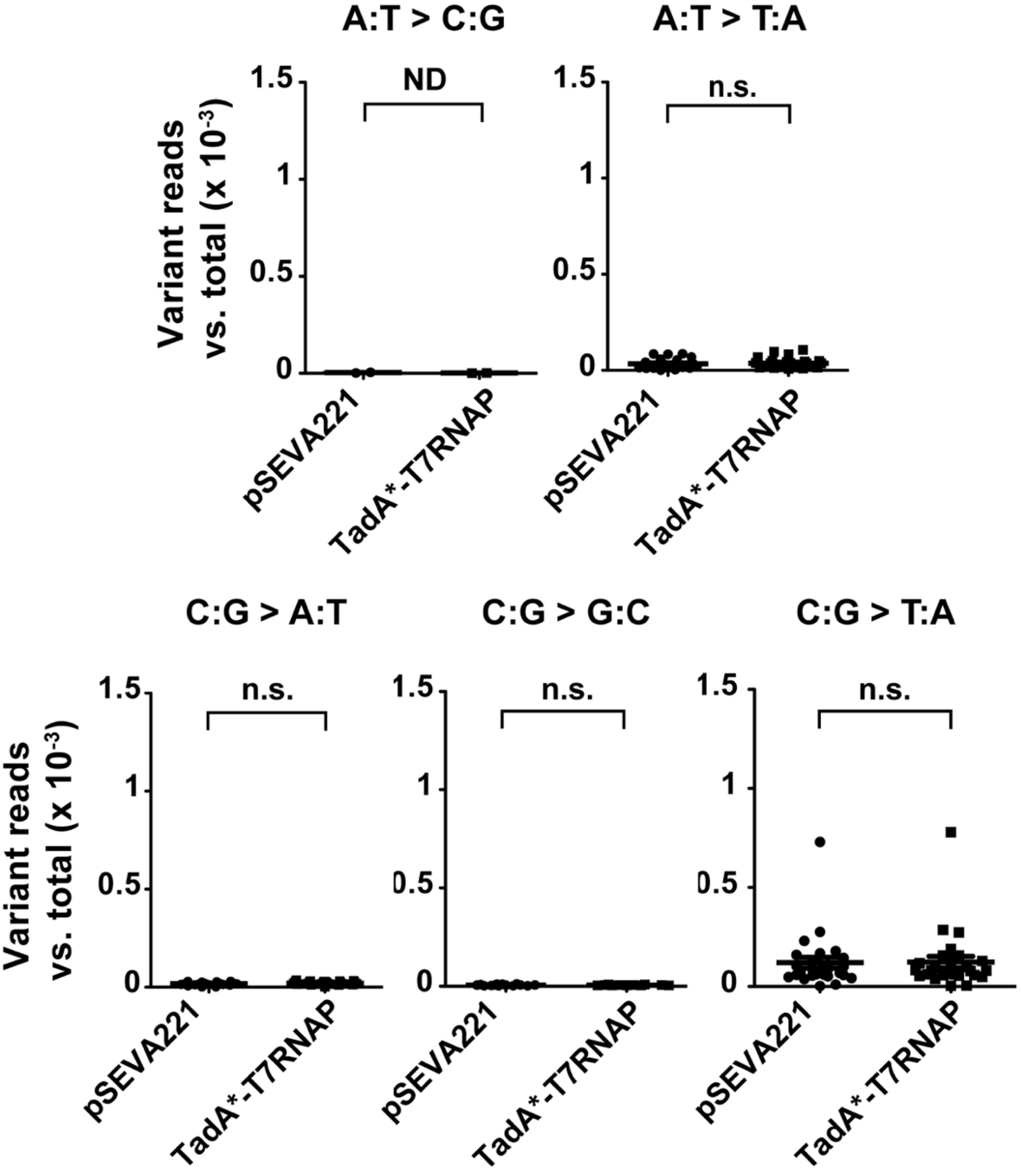

Supplementary Figure 7. High throughput sequencing of URA3 mutated with TadA*-T7RNAP. Variant calling analysis of a 200 bp region of URA3 after its massive DNA sequencing (ca. 10^6 reads) upon amplification from the strain MG*-URA3 *Δung Δnfi* with the empty plasmid (pSEVA221) or expressing TadA*-T7RNAP. The number of reads with different variants *vs*. total reads are represented with circles (empty plasmid) and squares (TadA*-T7RNAP). The number of variants found per group (n) in each graph was: A:T>C:G (pSEVA221 n=2, TadA*-T7RNAP n=2), A:T>T:A (pSEVA221 n=25, TadA*-T7RNAP n=24), C:G>A:T (pSEVA221 n=28, TadA*-T7RNAP n=27), C:G>G:C (pSEVA221 n=14, TadA*-T7RNAP n=13), C:G>T:A (pSEVA221 n=26, TadA*-T7RNAP n=26).The lines represent the means and the standard errors from each group. The statistical analysis was done using two-tailed Mann Whitney test. ND, not determined for groups with less than three variants detected, n.s, not significant. Source data are provided as a Source Data file.

Supplementary Figure 8

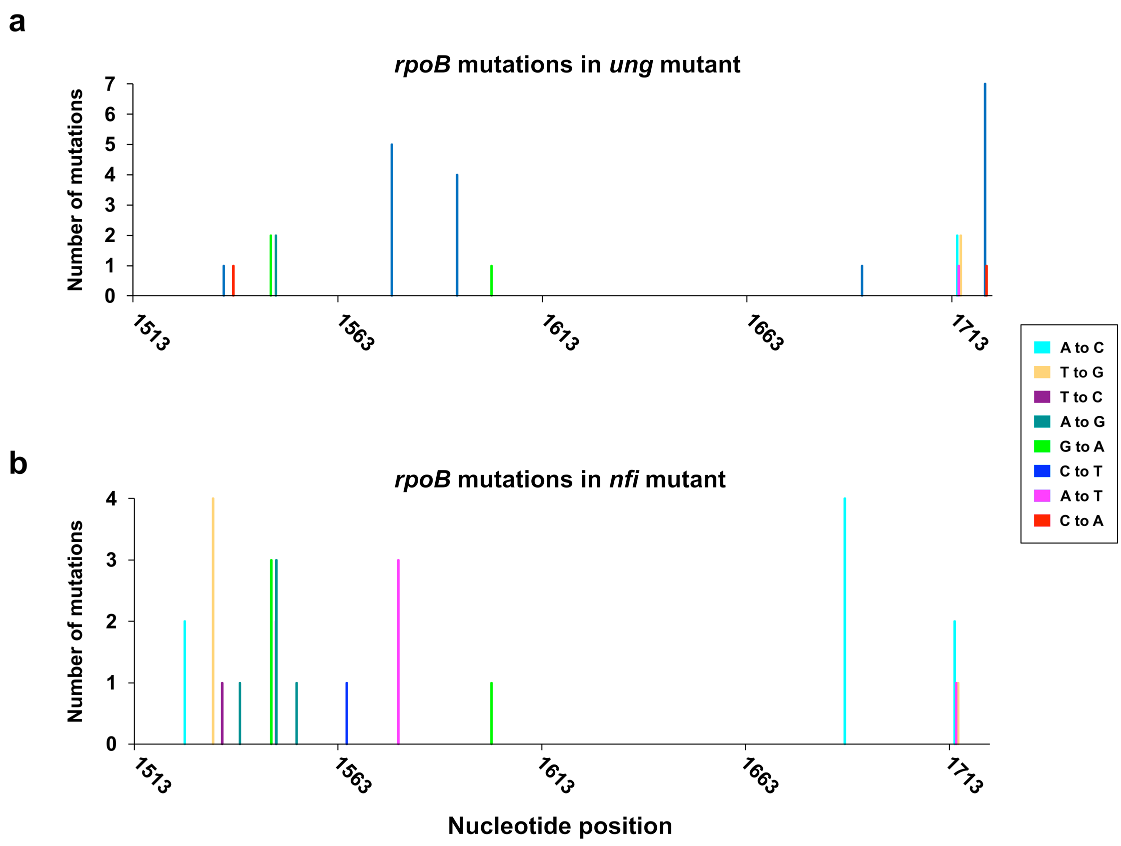

Supplementary Figure 8. Mutations in the *rpoB* gene from Rif^R colonies. Characterization of *rpoB* mutations in Rif^R colonies from the strains MG*-URA3Δ*ung* (**a**) and MG*-URA3Δ*nfi* (**b**) with the empty plasmid pSEVA221. For each culture, *rpoB* mutations from 30 colonies were determined The identified mutations (following the color code on the right) are indicated with respect to the coding strand of *rpoB*. Mutations were found within a 203 bp fragment of the *rpoB* gene corresponding to the nucleotides 1513-1716 (amino acids 505-572). Source data are provided as a Source Data file.

Supplementary Figure 9

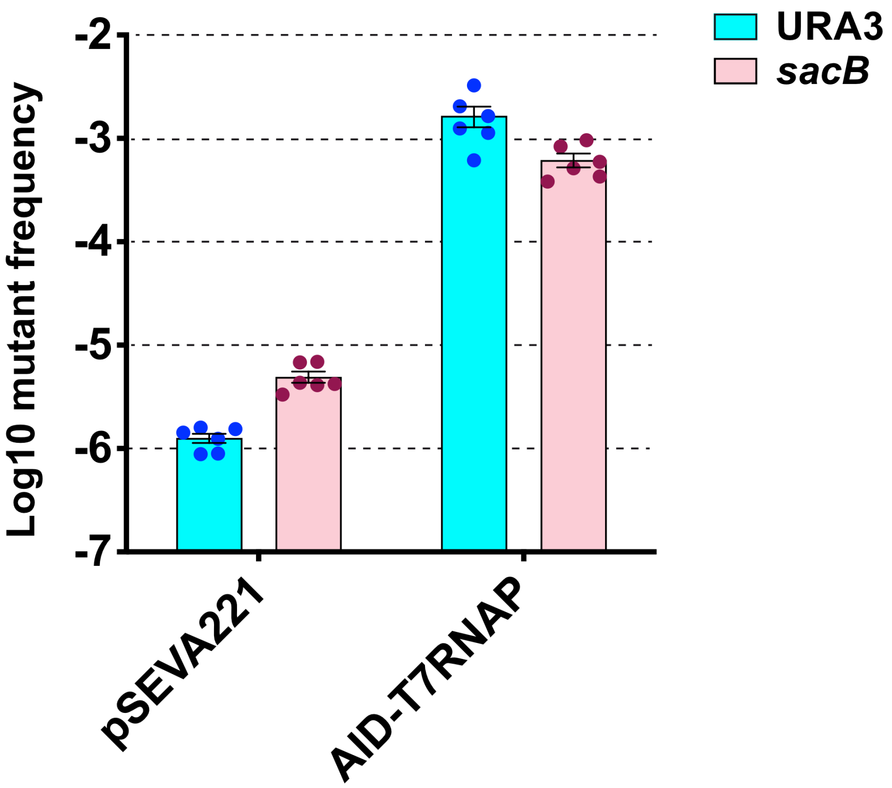

Supplementary Figure 9. Mutagenesis activity of AID-T7RNAP in the genes URA3 and *sacB*. Mutant frequency in *URA3* and *sacB* of the strain MG*-SacB-URA3Δ*ung*Δ*nfi* with pSEVA221 or expressing AID-T7RNAP. Single values from 6 independent experiments (n=6) are represented with color coded dots, means with bars and standard errors with black lines. Data Source data are provided as a Source Data file.

Supplementary Figure 10

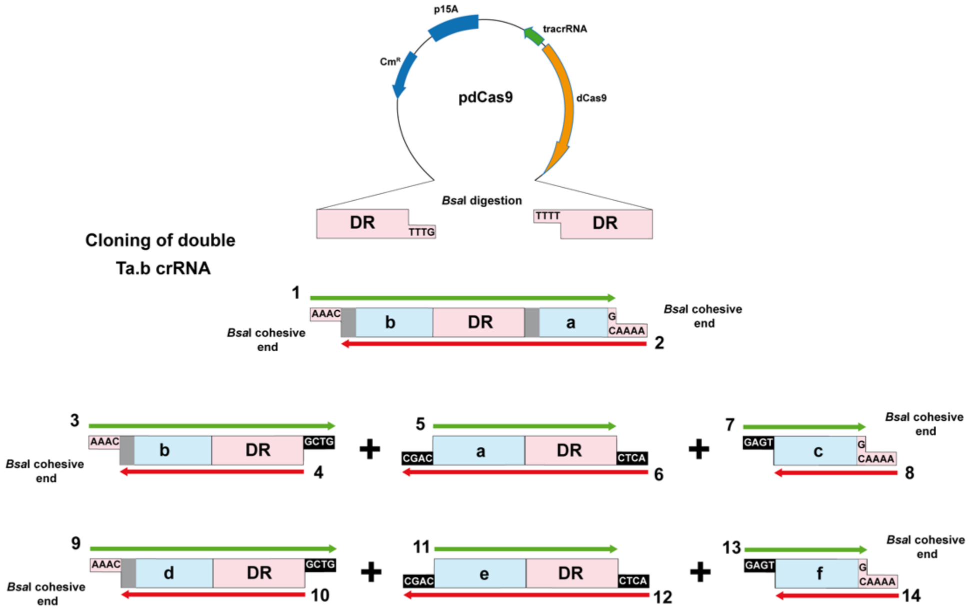

Supplementary Figure 10. Construction of pdCas9b.a, pdCas9b.a.c. and pdCas9d.e.f. Pink blocks represent direct repeats, blue blocks spacers and grey blocks extra sequences to complete the spacers. The complementary oligonucleotides are indicated with green and red arrows.

Supplementary Figure 11

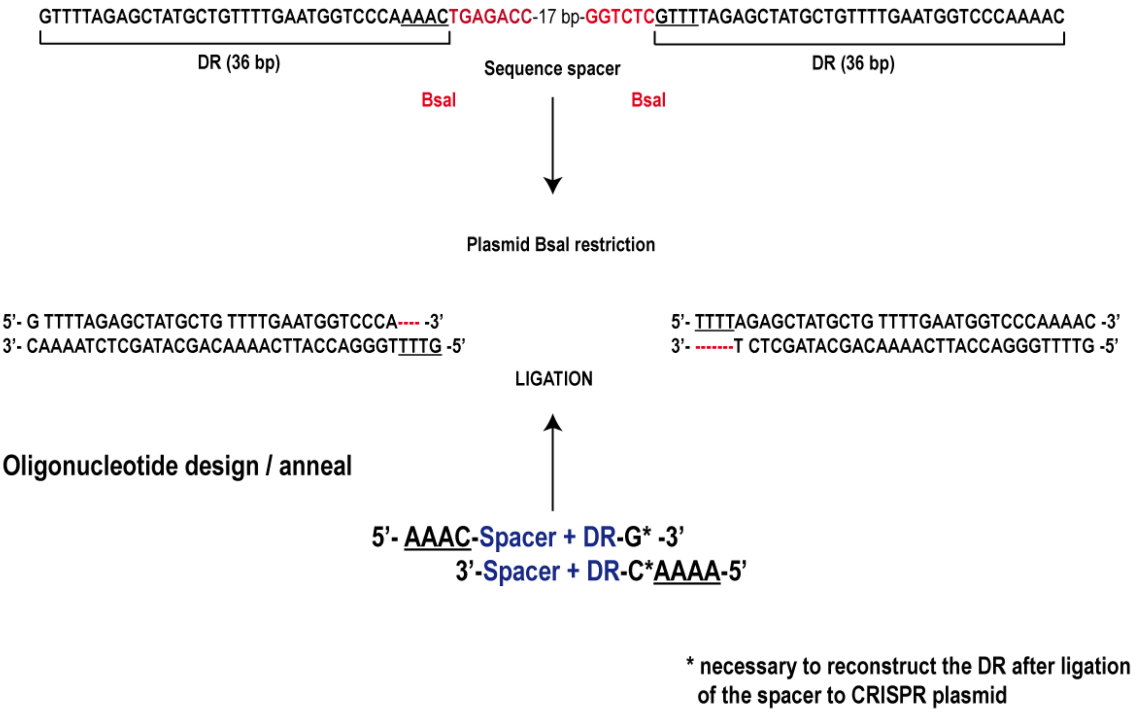

Supplementary Figure 11. Cloning the double and triple spacer arrays. Scheme showing the cloning strategy of double and triple crRNA arrays into the plasmid pdCas9 digested with *Bsa*I.

Supplementary references

1. Kern R, Malki A, Holmgren A, Richarme G. Chaperone properties of *Escherichia coli* thioredoxin and thioredoxin reductase. *Biochem J* **371**, 965-972 (2003).

2. Bach H*, et al.* Escherichia coli Maltose-binding Protein as a Molecular Chaperone for Recombinant Intracellular Cytoplasmic Single-chain Antibodies. *J Mol Biol* **312**, 79-93. (2001).

3. Bertram R, Hillen W. The application of Tet repressor in prokaryotic gene regulation and expression. *Microbial Biotechnology* **1**, 2-16 (2008).

4. Silva-Rocha R*, et al.* The Standard European Vector Architecture (SEVA): a coherent platform for the analysis and deployment of complex prokaryotic phenotypes. *Nucleic Acids Res* **41**, D666-675 (2013).

5. Severinov K, Soushko M, Goldfarb A, Nikiforov V. RifR mutations in the beginning of the Escherichia coli rpoB gene. *Molecular and General Genetics MGG* **244**, 120-126 (1994).

6. Petersen-Mahrt SK, Harris RS, Neuberger MS. AID mutates *E. coli* suggesting a DNA deamination mechanism for antibody diversification. *Nature* **418**, 99-103 (2002).

7. Sambrook J, Russel DW. *Molecular cloning. A laboratory manual*, 3rd edn. Cold Spring Harbor Laboratory Press (2001).

8. Piñero-Lambea C, Bodelón G, Fernández-Periáñez R, Cuesta AM, Álvarez-Vallina L, Fernández LA. Programming Controlled Adhesion of *E. coli* to Target Surfaces, Cells, and Tumors with Synthetic Adhesins. *ACS synthetic biology* **4**, 463-473 (2015).

9. Ruano-Gallego D, Álvarez B, Fernández LÁ. Engineering the Controlled Assembly of Filamentous Injectisomes in *E. coli* K-12 for Protein Translocation into Mammalian Cells. *ACS synthetic biology* **4**, 1030-1041 (2015).

10. Corcoran CP, Cameron AD, Dorman CJ. H-NS silences gfp, the green fluorescent protein gene: gfpTCD is a genetically Remastered gfp gene with reduced susceptibility to H-NS-mediated transcription silencing and with enhanced translation. *J Bacteriol* **192**, 4790-4793 (2010).

11. Li XT, Thomason LC, Sawitzke JA, Costantino N, Court DL. Positive and negative selection using the tetA-sacB cassette: recombineering and P1 transduction in *Escherichia coli*. *Nucleic Acids Res* **41**, e204 (2013).

12. Bolivar F, Rodriguez RL, Greene PJ, Betlach MC, Heyneker HL, Boyer HW. Construction and characterization of new cloning vehicles. II. A multipurpose cloning system. *Gene* **2**, 95-113. (1977).

13. Liao C*, et al.* Modular one-pot assembly of CRISPR arrays enables library generation and reveals factors influencing crRNA biogenesis. *Nature Communications* **10**, 2948 (2019).

14. Deltcheva E*, et al.* CRISPR RNA maturation by trans-encoded small RNA and host factor RNase III. *Nature* **471**, 602-607 (2011).

15. Jensen KF. The *Escherichia coli* K-12 "wild types" W3110 and MG1655 have an rph frameshift mutation that leads to pyrimidine starvation due to low pyrE expression levels. *Journal of Bacteriology* **175**, 3401-3407 (1993).

16. Datsenko KA, Wanner BL. One-step inactivation of chromosomal genes in *Escherichia coli* K-12 using PCR products. *Proc Natl Acad Sci U S A* **97**, 6640-6645. (2000).

17. Blattner FR*, et al.* The complete genome sequence of *Escherichia coli* K-12. *Science* **277**, 1453-1462 (1997).

18. Nyerges A*, et al.* A highly precise and portable genome engineering method allows comparison of mutational effects across bacterial species. *Proc Natl Acad Sci U S A* **113**, 2502-2507 (2016).

19. Herring CD, Glasner JD, Blattner FR. Gene replacement without selection: regulated suppression of amber mutations in *Escherichia coli*. *Gene* **311**, 153-163 (2003).

20. Martinez-Garcia E, Aparicio T, Goni-Moreno A, Fraile S, de Lorenzo V. SEVA 2.0: an update of the Standard European Vector Architecture for de-/re-construction of bacterial functionalities. *Nucleic Acids Res* **43**, D1183-1189 (2015).

21. Bikard D, Jiang W, Samai P, Hochschild A, Zhang F, Marraffini LA. Programmable repression and activation of bacterial gene expression using an engineered CRISPR-Cas system. *Nucleic Acids Res* **41**, 7429-7437 (2013).
