# Supplementary material for: In vivo diversification of target genomic sites using processive base deaminase fusions blocked by dCas9
Source: Nat Commun. 2020 Dec 22;11:6436. doi: 10.1038/s41467-020-20230-z (PMC7755918; doi:10.1038/s41467-020-20230-z)
Supplement: Supplementary file 2 — Reporting Summary [file 41467_2020_20230_MOESM2_ESM.pdf]

nature research

Corresponding author(s): Luis Ángel Fernández

Last updated by author(s): Nov 19, 2020

Reporting Summary

Nature Research wishes to improve the reproducibility of the work that we publish. This form provides structure for consistency and transparency in reporting. For further information on Nature Research policies, see Authors & Referees and the Editorial Policy Checklist .

Statistics

For all statistical analyses, confirm that the following items are present in the figure legend, table legend, main text, or Methods section.

n/a	Confirmed	
☐	☒	The exact sample size (*n*) for each experimental group/condition, given as a discrete number and unit of measurement
☐	☒	A statement on whether measurements were taken from distinct samples or whether the same sample was measured repeatedly
☐	☒	The statistical test(s) used AND whether they are one- or two-sided<br>*Only common tests should be described solely by name; describe more complex techniques in the Methods section.*
☒	☐	A description of all covariates tested
☒	☐	A description of any assumptions or corrections, such as tests of normality and adjustment for multiple comparisons
☐	☒	A full description of the statistical parameters including central tendency (e.g. means) or other basic estimates (e.g. regression coefficient) AND variation (e.g. standard deviation) or associated estimates of uncertainty (e.g. confidence intervals)
☐	☒	For null hypothesis testing, the test statistic (e.g. *F*, *t*, *r*) with confidence intervals, effect sizes, degrees of freedom and *P* value noted<br>*Give P values as exact values whenever suitable.*
☒	☐	For Bayesian analysis, information on the choice of priors and Markov chain Monte Carlo settings
☒	☐	For hierarchical and complex designs, identification of the appropriate level for tests and full reporting of outcomes
☒	☐	Estimates of effect sizes (e.g. Cohen's *d*, Pearson's *r*), indicating how they were calculated

Our web collection on statistics for biologists contains articles on many of the points above.

Software and code

Policy information about availability of computer code

Data collection	Massive sequencing data was collected with BaseSpace Sequence Hub version 5.28 software (Illumina Inc). In Flow Cytometry, data was collected with the software CXP Cytometer version 2.2 (Beckman Coulter Inc) and analysed with Kaluza Analysis version 2.1 (Beckman Coulter Inc).
Data analysis	Sequence reads obtained from Sanger chain-termination method was analysed with the program SeqMan Pro version version 12.3.1 (DNASTAR Lasergene). Obtained reads from NGS sequencing were processed with the program Bbmap version 28.36 for merging the paired-end reads. The resulting merged files were piled up against the reference sequence using the program Samtools version 1.9, and the variants were obtained with the program VarScan version 2.4.3 with the following parameters: --min-coverage 1 --min-reads2 1 --min-avg-qual 40 --min-var-freq 0.000001 --p-value 0.99. Statistic analysis was done with Prism 8.0 (GraphPad software Inc).

For manuscripts utilizing custom algorithms or software that are central to the research but not yet described in published literature, software must be made available to editors/reviewers. We strongly encourage code deposition in a community repository (e.g. GitHub). See the Nature Research guidelines for submitting code & software for further information.

Data

Policy information about availability of data

All manuscripts must include a data availability statement. This statement should provide the following information, where applicable:

- Accession codes, unique identifiers, or web links for publicly available datasets
- A list of figures that have associated raw data
- A description of any restrictions on data availability

Data that support the findings of this work can be found in the main manuscript and in the Supplementary information. Source data are provided with this paper. Figures with associated raw data are 2d, 2e, 3d, 3e, 3f, 4a, 4b, 4c, 4d, 4e, 4f, 5c, 5d, 6b, 7c; the Supplementary Figures 2d, 2e, 2f, 4, 6, 7, 8a, 8b and 9; the uncropped blots from the Figures 2b and 3b, and Supplementary Figure 2b with the information about the antibodies used and the working dilution; and the original uncropped images from the Supplementary Figure 1b. The sequences of the constructs built for this study are deposited in GenBank with the accession numbers and their hyperlinks listed in Supplementary Table 2. Sequencing data from highthrough-put DNA sequencing experiments are deposited in Sequencing

Read Archive (SRA) in the Bioproject ID PRJNA675288 (https://www.ncbi.nlm.nih.gov/bioproject/PRJNA675288). Materials and additional data are available from the corresponding author upon request.

Field-specific reporting

Please select the one below that is the best fit for your research. If you are not sure, read the appropriate sections before making your selection.

☒ Life sciences ☐ Behavioural & social sciences ☐ Ecological, evolutionary & environmental sciences

For a reference copy of the document with all sections, see nature.com/documents/nr-reporting-summary-flat.pdf

Life sciences study design

All studies must disclose on these points even when the disclosure is negative.

Sample size	No statistical methods were used to predetermine sample size. Data from at least three independent experiments (n≥3) was analysed because it is the standard in the field and the minimun size to determine statistical differences.
Data exclusions	No data were excluded from the analysis.
Replication	Data from at least three independent experiments was collected for analysis. All attempts at replication were successful.
Randomization	Individual colonies from each bacterial strain were selected randomly
Blinding	Investigators were not blinded for the experiments. Blinded is not required because colony counting on plates to calculate mutation frequencies is not a subjective parameter that could be influenced by the interpretation of the investigator.

Reporting for specific materials, systems and methods

We require information from authors about some types of materials, experimental systems and methods used in many studies. Here, indicate whether each material, system or method listed is relevant to your study. If you are not sure if a list item applies to your research, read the appropriate section before selecting a response.

Materials & experimental systems

n/a	Involved in the study	
☐	☒	Antibodies
☒	☐	Eukaryotic cell lines
☒	☐	Palaeontology
☒	☐	Animals and other organisms
☒	☐	Human research participants
☒	☐	Clinical data

Methods

n/a	Involved in the study	
☒	☐	ChIP-seq
☐	☒	Flow cytometry
☒	☐	MRI-based neuroimaging

Antibodies

Antibodies used	For detection of the BD-T7RNAP fusions, monoclonal mouse anti-T7 RNA polymerase antibodies at a dilution of 1:10000 (Novagen, Merck; ref 70566-3, kit batch number D00143325) and secondary peroxidase-labelled goat anti-mouse antibodies at a dilution of 1:5000 (Sigma A2554) were used.
Validation	The monoclonal mouse anti-T7 RNA polymerase antibodies were validated by the commercial supplier as stated on Merck website: "The T7 RNA Polymerase Monoclonal Antibody is a mouse monoclonal antibody (IgG1) that specifically recognizes T7 RNA polymerase. It can be used to monitor the expression of T7 RNA polymerase in bacterial and eukaryotic systems" Specificity: Bacteriophage T7 RNA polymerase, residues 861–883 (C-terminus), native and denatured enzyme. Cross-reactivity: Negligible with bacterial, insect, or mammalian cell lysates. Sensitivity: < 12.5 ng (Western blot developed with chromogenic substrates). Working dilution: 1:10,000 for Western blotting

Flow Cytometry

Plots

Confirm that:

- [x] The axis labels state the marker and fluorochrome used (e.g. CD4-FITC).
- [x] The axis scales are clearly visible. Include numbers along axes only for bottom left plot of group (a 'group' is an analysis of identical markers).
- [x] All plots are contour plots with outliers or pseudocolor plots.
- [x] A numerical value for number of cells or percentage (with statistics) is provided.

Methodology

Sample preparation	The volume corresponding to one unit of optical density (O.D.) at 600 nm of the bacterial induced cultures was collected by centrifugation (3300xg, 5 min) and resuspended in 0.5 ml 1X PBS. The cell suspension was diluted transferring 0.2 ml to a tube with 1.2 ml of 1X PBS, and its fluorescence levels was determined using a Gallios FC500 flow cytometer (Beckman Coulter).
Instrument	Gallios FC500 flow cytometer (Beckman Coulter Inc)
Software	Data was collected with the software CXP Cytometer version 2.2 (Beckman Coulter Inc) and analysed with Kaluza Analysis version 2.1 (Beckman Coulter Inc)
Cell population abundance	Fluorescence of aprox. 100,000 events (bacterial cells) for each population was determined
Gating strategy	Bacteria were gated with a log representation of FSC vs SSC, selecting size corresponding to bacterial cells as shown in the Supplementary Figure 3

- [x] Tick this box to confirm that a figure exemplifying the gating strategy is provided in the Supplementary Information.
